# MIT Toxicogenomics Research Program

**DOI:** 10.1289/ehp.113-a234

**Published:** 2005-04

**Authors:** Mary Eubanks

The Toxicogenomics Research Consortium (TRC) was initiated by the NIEHS to integrate genomics approaches into the field of toxicology through the cooperative efforts of research institutions from academia, government, and industry. At one TRC member institution, the Massachusetts Institute of Technology (MIT), researchers are focusing their attention on the effects of simple and complex alkylating agents, a class of DNA-damaging environmental toxins and toxicants that cause cell death, mutations, birth defects, and cancer.

The MIT Toxicogenomics Research Program was established in September 2001 and is directed by Leona D. Samson, a professor of toxicology and biology engineering and director of the MIT Center for Environmental Health Sciences. In the short time since the first TRC grants were awarded, the MIT program has made numerous significant contributions to toxicogenomics in scientific discovery, innovation, collaboration, and education.

## Alkylating Agents

Alkylating agents are highly reactive chemicals that introduce alkyl groups into biologically active molecules and prevent normal functioning. They are found in the environment and are produced within the body during metabolism—we are, in fact, continuously exposed to these agents. Most alkylating agents are extremely toxic; some are used for chemotherapy.

Samson is interested in pathways that repair DNA alkylation damage, and any other pathways that ameliorate or exacerbate their toxicity. One aspect of her research focuses on expression of genes as a result of exposure to alkylating agents. Assaying gene expression using microarray technology, her lab has identified thousands of genes whose transcript levels are altered as a result of exposure.

Surprisingly, it is not only DNA repair and cell cycle checkpoint genes that are transcriptionally regulated upon exposure to alkylating agents, but also genes involved in many unexpected pathways such as protein, RNA, and lipid metabolism. This was first demonstrated in *Saccharomyces cerevisiae*, and the Samson lab is currently exploring whether such a broad response is evolutionarily conserved. One current topic of investigation is the transcription response of mice with different DNA alkylation repair capabilities upon exposure to alkylating agents.

Samson’s results challenge some of the basic concepts of how we think about the response of cells to DNA-damaging agents. Further, they reveal that responses to environmental insult are much more complex than previously thought. It has become clear that a global systems model is a more accurate way of viewing toxicity. This insight into the complexity of environmental exposure responses led to the incorporation of the Toxicogenomics Research Program into the school’s Center for Environmental Health Sciences.

## Understanding Aflatoxin

John Essigmann, a professor of chemistry, toxicology and biological engineering, conducts research on differential gene expression in response to exposure to aflatoxin, a natural DNA-damaging agent that is widespread in the environment. Aflatoxin is a fungal toxin produced by *Aspergillus flavus*. Acute liver toxicity and liver cancer are common features of people exposed to aflatoxin-contaminated food, particularly peoples in developing areas where cereal grains are the staple food in the diet. Such areas include sub-Saharan Africa, China, India, and Southeast Asia, where the diet includes lots of rice, peanuts, and maize. In areas of the world where aflatoxin contaminates the food supply, there is concern that children, especially, are compromised in their growth and overall health.

People infected with hepatitis B have increased sensitivity to aflatoxin carcinogenesis by as much as 50- to 100-fold. Aflatoxin and hepatitis B each are major risk factors for liver cancer (more so when combined), a leading cause of cancer death in Thailand, among other parts of the world. Essigmann was recently awarded Thailand’s Princess Chulabhorn Gold Medal for his “commitment to and sustained support for the advancement of science in developing countries, as well as for his selfless dedication to teaching and research.” In the developing world, it is difficult to completely eliminate aflatoxin from the diet, so Essigmann has emphasized that it is essential to promote hepatitis vaccination for children, as this disease is the co-etiologic factor in the prevalence of liver cancer in these areas.

The Essigmann lab is working to understand the mechanisms of toxicity and liver disease associated with aflatoxin exposure. Aflatoxin reacts with guanine bases in DNA to form adducts or lesions that can cause errors in DNA replication; these errors presumably lead to malignancy. Aflatoxin can form many different types of adducts, and different organisms vary in their response to exposure. Some species, such as rats, are highly sensitive and susceptible, whereas others, such as mice, are resistant. Essigmann is particularly interested in the reasons for the differential sensitivity of different species to aflatoxin, as well as the reasons that hepatitis amplifies human risk of aflatoxin carcinogenesis.

With respect to the first question, Essigmann has compared the sensitivity of adult and infant mice to aflatoxin. Although adult mice are resistant to liver cancer induced by the fungal toxin, infant mice are exquisitely sensitive. Four-day-old mice exposed to aflatoxin develop cancer, but by one week of age, they become resistant. By investigating differences in the gene expression patterns of mice exposed at different developmental stages, Essigmann hopes to find clues to better understand why some people are sensitive and some are resistant, how race and gender may affect why some people respond to therapeutic drugs and others do not, why infants and children are more susceptible than adults, and why hepatitis confers heightened sensitivity to aflatoxin effects.

## Liver on a Chip

Linda Griffith, a professor of biological engineering and mechanical engineering and director of the Biotechnology Process Engineering Center at MIT, was voted one of the annual “Brilliant 10” young scientists by *Popular Science* in 2002 for her ground-breaking research in tissue engineering and her vision for creating the human body on a chip. With funding from the Defense Advanced Research Project Agency, the central research and development agency of the Department of Defense, Griffith designed a prototype “liver on a chip.” The liver is highly sensitive to all kinds of environmental toxicants, and the objective of the initial work was to develop a tiny, portable biosensor that could be used in battlefield deployment for early detection of environmental hazards from chemical and biological warfare. Continuing support for further development of the liver chip for sensing applications is provided by the Institute for Soldier Nanotechnologies at MIT. Among other valuable applications for the liver on a chip is *in vivo* experiments in toxicogenomics.

The liver on a chip comprises liver cells affixed to tiny channels etched in a silicon chip that is smaller than a quarter and housed in a clear plastic watertight housing. The tiny reactor holds up to 50,000 liver cells distributed among 40 identical units that each resemble the capillary bed, or network, of a tissue. The system can readily be scaled up to contain over a million cells in a larger reactor with 1,000 tiny “capillary beds.” Within the bioreactor, oxygen- and nutrient-enriched fluid that mimics the blood supply flows through the silicon scaffold, simulating the body’s circulation system. This miniature three-dimensional (3-D) model of the liver maintains the enzymes essential for metabolizing toxicants longer and performs better than two-dimensional cell culture *in vitro* systems.

The 3-D microscale liver bioreactor is an important new toxicology tool for examining *in vivo* gene expression in primary liver cells in response to acute and chronic exposure to environmental toxins and other toxicants. With the validation of optimal levels of gene expression of enzymes in toxin metabolism, Griffith’s lab is starting to employ the liver bioreactor to profile effects of toxins on liver metabolism. Initial experiments are looking at the response of rat liver cells to aflatoxin exposure. Aflatoxin is added to the circulating fluid, and the cells are subsequently examined microscopically to detect what is happening in the tissue. Preliminary results, reported at the TRC biannual meeting in December 2004, indicate there is a dose-dependent relationship between exposure and toxicity.

Cell cultures, which generally are flat and one cell thick, have a limited capacity to reproduce *in vivo* cellular function. The 3-D bioreactor provides a better simulation of living tissue and real liver function. Liver cells grown in the artificial structure replicate and clump together to form a complex extracellular matrix. This highly evolved microenvironment maintains cellular signaling. The liver bioreactor makes it possible to study the complex order of tissue in a system that can be manipulated by drugs, hormones, and environmental toxins. Early experiments are demonstrating appropriate levels of gene expression, especially enzymes in toxin metabolism.

Griffith is just beginning to test the bioreactor’s capacity to profile toxins involved in liver metabolism. Ultimately, she wants to conduct experiments with human cells as an accessible way to look at human responses to exposure.

## A Group Effort

In addition to the three independent investigator-initiated projects of Samson, Essigmann, and Griffith, the MIT scientists participate as collaborators on three projects involving investigators from TRC cooperating research members (CRMs) as well as a consortiumwide microarray standardization effort.

The first project is a collaboration with Oregon Health & Science University, the Fred Hutchinson Cancer Research Center, the University of Washington, and the National Center for Toxicological Research to examine the roles of DNA alkylation in neurodegenerative disease and cancer. Compounds being tested include methyl-methoxymethanol, which comes from the naturally occurring toxin cycasin (from a plant in the cycad family), as well as *N*-methyl-*N*-nitrosourea and methyl methane sulfonate, both of which are synthetic compounds found in grilled foods and smoke.

The second project is a collaboration with Duke University to identify a common gene expression profile indicative of stress response across organisms that include yeast, the worm *Caenorhabditis elegans*, and humans. The compounds tested thus far include *N*-methyl-*N*′-nitro-nitrosoguanidine (an alkylating agent similar to methyl methane sulfonate) and cadmium (a toxic metal found in tap water). Test data are being analyzed to identify signature profiles for these toxicants, identify transcriptional genes that are conserved, and define conserved regulatory pathways that control the response.

The third project is a collaboration with the University of North Carolina at Chapel Hill and the NIEHS to examine nuclear receptor–mediated toxicity and oxidative damage in mouse and human cells in the liver bioreactor. Knockout mice that have deletions in two different nuclear receptors are being tested to see if responses to toxicants are mediated by those receptors.

Fundamental to these projects as well as to the independent research projects of the MIT CRM and other TRC members is the quality of the gene expression data. An overarching goal of the TRC is cross-platform, cross-laboratory comparison of gene expression profiling. Each participating member has contributed results from a standard experiment to a meta-analysis. MIT’s share of the data was coordinated by Rebecca Fry, a research scientist in computational and systems biology and assistant director of the MIT Microarray and Bioinformatics Center. Fry analyzed the microarray data for meaningful biological interpretation and standardization. A meta-analysis of around 800 data sets from a consortiumwide microarray study of two standardized biological samples revealed poor correlation across laboratories and between platforms. This provided the basis for recommendations for common steps in data processing to improve data comparability between platforms and investigators. Fry presented these data at the 2004 TRC biannual conference.

The MIT Toxicogenomics Research Program is making considerable contribution to the field of toxicogenomics. Through the application of new computational tools to integrate high-quality data sets including phenotypic responses to alkylation damage and known protein–protein interactions, the Samson lab has illustrated that interacting networks of proteins are essential for recovery after damage. These applications of systems biology have enabled MIT to play a strong leadership role in expanding the horizons in toxicogenomics. With these broadened horizons we can expect to ultimately see improved assessment and predictive powers for environmental health, new therapies and biomarkers for exposure, and environmental policies that are based on more accurate scientific knowledge.

## Figures and Tables

**Figure:**
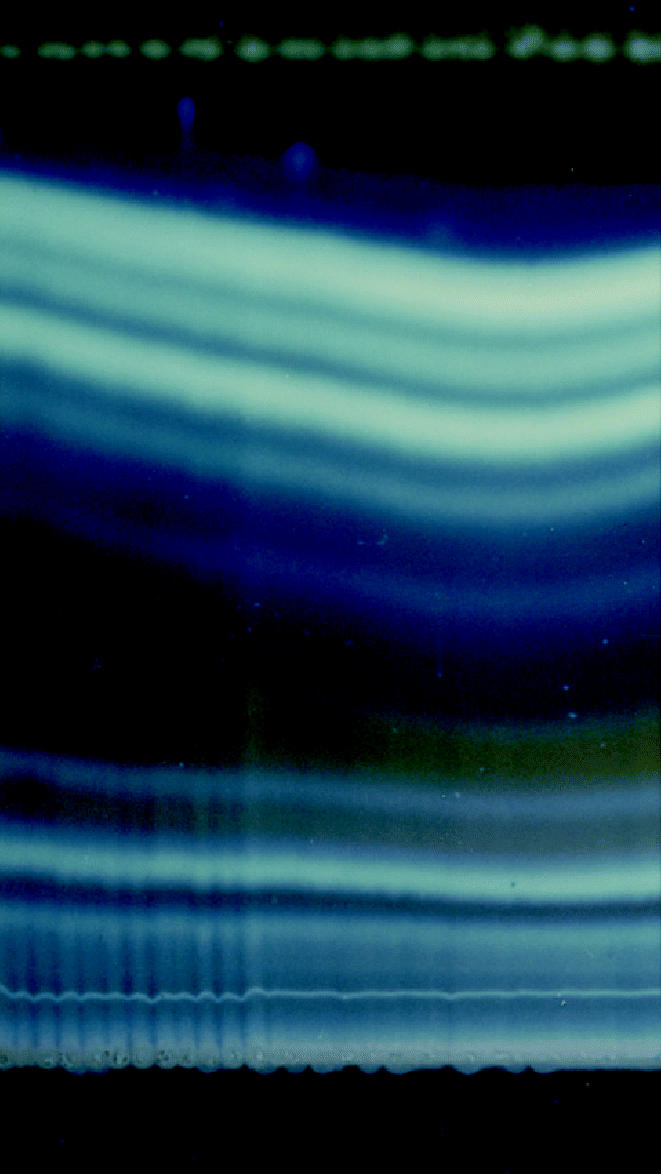

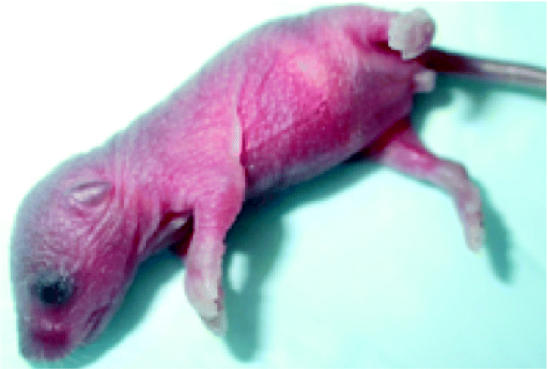
**Solving a mechanistic mystery.** Researchers are studying aflatoxin, a food contaminant that fluoresces blue under UV light (above), to understand genetic susceptibility. Intriguingly, aflatoxin causes liver cancer in 4-day-old mice (right), but week-old mice are resistant.

